# “See You Later, Alligator”: A Case of Anaphylaxis to Alligator Meat Consumption

**DOI:** 10.7759/cureus.77226

**Published:** 2025-01-10

**Authors:** Vickie Xin, Robert Leone, Devang Doshi

**Affiliations:** 1 Internal Medicine, Corewell Health William Beaumont University Hospital, Royal Oak, USA; 2 Allergy and Immunology, School of Medicine, Wayne State University, Detroit, USA; 3 Allergy and Immunology, Corewell Health William Beaumont University Hospital, Royal Oak, USA

**Keywords:** allergy, alligator allergy, anaphylaxis, chicken allergy, fish allergy, reptile allergy

## Abstract

*Alligator mississippiensis* meat is an uncommon, exotic dish that has increasingly become easily accessible across the United States. This presents a unique issue for patients with food allergies, as the cross-reactivity of reptile meat with fish and poultry allergy is not well-recognized. While there has been an established connection between those with fish allergy also reacting to reptile meat, it is even less known that poultry allergy may also have cross-reactivity with reptile meat. We present a case of a 25-year-old male patient who is able to consume fish but is allergic to chicken and who consumed alligator meat twice. During the second exposure, the patient experienced an anaphylactic reaction, as he was unaware of the possible cross-reactivity with his poultry allergy.

## Introduction

Allergy to reptile meat, specifically crocodile and alligator, has been documented in the setting of known fish allergy [1,2]. However, there are fewer cases of allergy to alligator meat in the setting of known chicken allergy. Studies through molecular analysis have shown links between alligator meat to fish and chicken through the parvalbumin protein [3-5], which is a calcium-binding protein [5]. Easier access to alligator and crocodile meat presents a unique challenge particularly for fish- and chicken-allergic patients, as it is not commonly known that there is cross-reactivity with reptile meat. While statistics on alligator meat consumption are scarce, Louisiana is one of the few states where harvesting alligator meat is legal and conducts close monitoring of the hunting season [6]. The Louisiana Wildlife and Fisheries Department reported that in 2022, a total of 23,000 alligators were harvested with an estimated value of nearly $5.5 million [6]. In some instances, patients may opt to consume reptile meat as their protein source in light of their fish or chicken allergy; however, it is not well-recognized that cross-reactivity and life-threatening anaphylaxis may occur with consuming reptilian meat. This case highlights the importance of educating our patients on the possibility of cross-reactivity as exotic meats become more available.

## Case presentation

A 25-year-old male patient with a past medical history significant for eosinophilic esophagitis presented for follow-up after being treated in the emergency room for an anaphylactic reaction to eating alligator meat. The patient lives in southeast Michigan and originally sourced the exotic meat from a butcher in the area. The patient’s additional allergic profile included chicken, turkey, legumes, soy, and eggs. He was able to consume fish without an allergic reaction.

The patient initially carefully prepared alligator meat in his home during his first exposure and subsequently developed mild throat discomfort. He initially believed these symptoms to be a flare of his eosinophilic esophagitis. The throat discomfort was subsequently relieved following two doses of budesonide oral suspension. His initial symptoms of dysphagia and throat irritation slowly subsided after home medication. For this reason, he did not seek our further medical intervention. Approximately one week following the first exposure, he consumed the leftover alligator meat and, within five minutes, developed facial flushing, throat swelling, and shortness of breath. He self-administered two doses of subcutaneous epinephrine, oral diphenhydramine, and albuterol. He was taken to the emergency department and, there, received additional intravenous (IV) diphenhydramine and steroid injections. He was observed for four hours and then discharged home. The patient was then seen at his allergist's office for follow-up and advised to discontinue consuming alligator or crocodile meat given the chance of cross-reactivity. 

**Table 1:** Allergic profile

Allergic food panel	
Fish (0.00-0.34 kU/L)	<0.35 kU/L
Chicken (0.00-0.34 kU/L)	1.79 kU/L
Turkey (0.00-0.34 kU/L)	0.47 kU/L
Peanut (0.00-0.34 kU/L)	26.10 kU/L
Egg white (0.00-0.34 kU/L)	3.84 kU/L
Soybean (0.00-0.34 kU/L)	1.32 kU/L
Immunoglobulin E (<100 IU/mL)	127 IU/mL

## Discussion

Alligator meat is a common dish in the southeastern United States. In the last decade, it has become more easily accessible nationally, as demonstrated by this patient sourcing the meat from a butcher in southeast Michigan. Prior to the patient consuming the alligator meat, he was unaware of any potential cross-reactivity with his known chicken allergy. 

When studying reptilian meat allergy, the main area of focus has been its cross-reactivity with fish. The most-studied allergenic protein has been the parvalbumin protein, a calcium-binding protein [1-5]. This protein is often found in fast-twitch skeletal muscles and the nervous system and is often the cause of fish allergy [2]. It has been shown through molecular analysis that the alpha- and beta-isoforms of parvalbumin are the main proteins involved in fish and reptile allergy cross-reactivity given their similarity in protein structure, with beta-parvalbumin considered as the main immunoglobulin E-binding allergen [5]. 

On the other hand, while the cross-reactivity of fish and reptile allergy have been studied thoroughly, Ballardini et al. were the first to report a chicken and reptile meat cross-reactivity [1]. Similar to the case reported by Ballardini et al., our patient had a known chicken and turkey allergy and reacted to consuming reptile meat [1]. Molecular analysis conducted showed that the alpha-parvalbumin was similar between the chicken and reptile meat [1]. A previous study also found fish and chicken cross-reactivity and attributed parvalbumin, enolaces, and aldolases as allergenic proteins [3]. To the best of our knowledge, this patient is only the second reported case of an anaphylactic reaction to reptilian meat in the setting of known poultry allergy but in the absence of fish allergy. With his history, it is likely he is allergic to the alpha-parvalbumin protein. 

**Table 2:** Summary of cross-reactions and the causal allergenic protein

Cross-reaction	Allergenic protein
Fish x reptile	Beta-parvalbumin
Chicken x reptile	Alpha-parvalbumin
Fish x chicken	Parvalbumin, enolases, aldolases

Further investigation into meat processing found that varying histamine levels are present in meats [7]. The levels will differ based on processing, type, and age of the meat. A study performed by the Hallym University College of Medicine showed increased histamine levels based on cooking methods. Frying and grilling have the largest increase in histamine levels [8]. The patient both fried the alligator as well as consumed it days later, which could have played a role in the exacerbation of an anaphylactic reaction. Interestingly, pork and anchovies were the meats with the highest histamine concentration, not chicken or other parvalbumin-containing fish [7]. While cross-reactivity to alpha-parvalbumin is plausible given this patient's allergic profile, histamine content in the processing of alligator meat must also be considered. 

Evolution-based genomic studies have revealed a close relationship between birds and crocodiles; both species belong to a monophyletic group called the archosaurs [9]. The evolutionary similarity of these species could provide the basis for cross-reactivity between avians, crocodiles, and alligators. This could also support why both types of meat contain similar parvalbumin, as close evolutionary species would have similar, if not the same, conserved genomic sequences and, thus, proteins. 

## Conclusions

This case demonstrates that patients with allergies to poultry can be cross-sensitized to reptile meat and have anaphylactic reactions to consuming reptile meat. The presence of parvalbumin in both alligator and crocodile meat can pose a serious immunologic threat to those not only with fish allergies but severe chicken allergies as well. Without readily available testing for reptile meat, it is crucial to advise patients with a history of either poultry or fish allergy of the possibility of cross-reactivity. Future studies should be conducted for the cross-reactivity of exotic meats with the increased accessibility and use of these meats. 
